# Evaluation of iodine detection and quantification limits in photon-counting CT—a phantom study

**DOI:** 10.1186/s41747-026-00824-w

**Published:** 2026-10-12

**Authors:** Rebecca Titternes, Gavin Poludniowski, Torkel Bo Brismar, Patrik Nowik, Daniel Thor

**Affiliations:** 1 Department of Nuclear Medicine and Medical Radiation Physics, Karolinska University Hospital, Stockholm, Sweden; 2 Department of Clinical Science, Intervention and Technology, Karolinska Institutet, Stockholm, Sweden; 3 Department of Radiology, Karolinska University Hospital, Huddinge, Sweden; 4 Siemens Healthineers, Stockholm, Sweden; 5 Department of Oncology-Pathology, Karolinska Institutet, Stockholm, Sweden

**Keywords:** Contrast media, Iodine, Limit of detection, Quantification, PCCT

## Abstract

**Objective:**

Clinical decisions using CT iodine maps are based on evaluation of contrast enhancement, making it essential to understand how accurately CT systems detect and quantify iodine, yet quantitative evaluations remain scarce, and few studies treat iodine as an analyte for which detection threshold, quantification precision, and measurement linearity can be determined using standard laboratory-science concepts. No study has applied such concepts across photon-counting CT (PCCT) and dual-energy CT (DECT) systems.

**Materials and methods:**

Using head and abdominal protocols, test tubes with iodine concentrations (0.0–2.0 mg/mL) were placed in a phantom providing head- and abdomen-equivalent attenuation. Scans were performed at two dose levels per protocol, on two DECT and one PCCT system. Iodine images were reconstructed for repeated scans using varying iterative reconstruction (IR) levels. Limit-of-detection (LoD), limit-of-quantification (LoQ), and lower-limit-of-linear-interval (LLLI) were calculated for two ROI sizes (3 and 10 mm), and differences between scanners were analyzed with Welch ANOVA and Games–Howell tests (α = 0.05).

**Results:**

PCCT demonstrated significantly lower LoD/LoQ values than the DECT systems across most protocols. For low-dose abdomen (3 mGy, medium-IR), LoD for 3 and 10 mm ROI size were 0.78 and 0.16 mg/mL (PCCT), 1.28 and 0.26 mg/mL (split-filter CT), and 1.42 and 0.57 mg/mL (dual-source CT). LoQ values were also lower for PCCT. Improvements were most pronounced in low-dose protocols, with LoD reductions up to 68% (brain) and 45% (abdomen). LLLI was similar across systems.

**Conclusion:**

PCCT outperformed DECT in iodine detection and quantification, particularly in low-dose protocols. These findings highlight the potential use of reduced iodine contrast and/or radiation dose.

**Key Points:**

***Question*** Objective comparison of iodine detection and quantification performance between PCCT and DECT systems remains limited, particularly for low-iodine contrast media doses.

***Findings*** PCCT achieved significantly lower detection and quantification thresholds than conventional CT systems, particularly for small lesions and low-dose imaging protocols.

***Relevance statement*** Task-based LoD and LoQ metrics enable objective comparison of CT system performance and may provide clinically relevant thresholds for iodine detectability, quantification, and protocol optimization.

**Graphical Abstract:**

**Figure:**
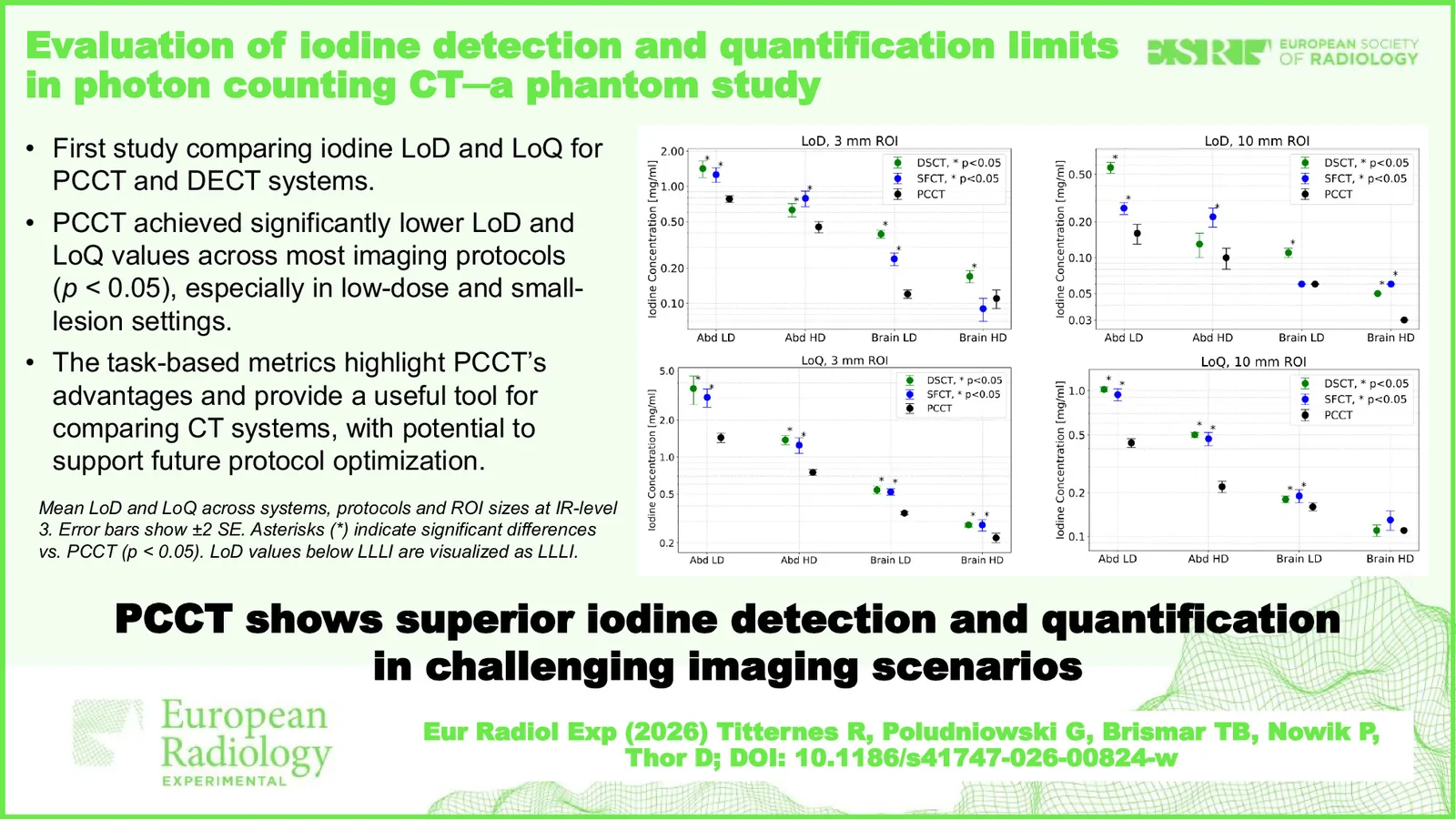

## Background

Computed tomography iodine maps are widely used in neuro- and abdominal imaging to quantify iodine uptake, support lesion characterization, distinguish contrast extravasation from hemorrhage, identify subtle lesion enhancement, and separate iodine from other hyperdense findings [1–4]. Iodine maps are often used as a problem-solving tool when non-contrast phases are missing or when small differences in enhancement are diagnostically important. Until recently, the generation of iodine maps has required dual-energy CT (DECT) systems [5–10] such as dual-source CT (DSCT), split-filter CT (SFCT), or dual-layer detectors, which create low- and high-energy spectra, using dual X-ray tubes, beam-splitting filters, or layered detectors, respectively [11–13]. DECT is based on energy-integrating detector (EID) technology. Photon-counting CT (PCCT) instead represents a major advancement in CT imaging technology, offering reduced image noise, improved contrast-to-noise ratio (CNR), more stable Hounsfield units (HU) at low radiation doses, with enhanced spatial resolution [14–18].

PCCT counts individual photons and distinguishes their energy levels, enabling the creation of discrete energy bins. In addition, it provides access to multi-energy data in every scan (“always on”) and offers the potential for improved tissue characterization and visualization of contrast agents [11, 17, 19–23].

In current practice, iodine maps are frequently used in conjunction with concentration thresholds [24]. Therefore, a central question in quantitative iodine imaging is whether a measurement is trustworthy; understanding the accuracy and precision of reconstructed concentration is vital. Simple and commonly used image quality metrics such as CNR are based on assumptions about noise properties and reconstruction algorithms that may limit their suitability for quantitative comparison across CT systems or reconstruction methods, particularly when spatial resolution or noise texture differ [25, 26]. Another commonly used approach is model observer methods, which, although able to overcome these shortcomings, typically focus on lesion detectability rather than quantitation of an elevated signal within a region [27]. There is a need to answer clinically relevant questions, such as: (1) Can faint iodine enhancement in a region be detected through ROI measurements? (2) At what concentration is an ROI-based measurement precise enough to be reliable? and (3) Over what range of concentrations does the system behave linearly with true iodine concentration? To address this, we apply three analytical metrics to iodine maps. Limit of detection (LoD) is the lowest iodine concentration distinguishable from the background, indicating the concentration at which subtle iodine enhancement can first be separated from the background level. Limit of quantification (LoQ) is the lowest concentration at which an ROI-based measurement is quantitatively reliable, relevant when subtle enhancement differences influence diagnosis or follow-up. Lower limit of linear interval (LLLI) is the lowest concentration at which iodine measurements remain proportional to true iodine content, important for low-iodine levels and contrast-media saving protocols. These metrics are well-established for evaluating the performance of systems that determine analyte concentration, particularly in clinical chemistry and molecular diagnostics [28], and the methodology is outlined in the Clinical and Laboratory Standards Institute (CLSI) EP17-A2 and EP06 guidelines [29–31]. Recently, it was extended to DECT iodine imaging by Jacobsen et al [32, 33]. By quantifying how ROI measurements vary across repeated scans, they directly assessed the robustness of iodine quantification in practice.

In this study, we compare LoD, LoQ, and LLLI between two DECT systems (SFCT and DSCT) and one PCCT system using clinically representative brain and abdominal protocols, further refining the application of the methodology to CT. This is the first study to evaluate these quantitative performance limits in a PCCT system. The presented framework provides clinically interpretable thresholds for iodine detectability, measurement reliability, and linearity, supporting objective comparison of different CT technologies. The contexts of small and large region sizes and low and high radiation doses are investigated, with relevance to imaging protocols with low contrast-media concentrations.

## Methods

LoD, LoQ, and LLLI provide direct, concentration-based thresholds for iodine detection, quantification, and linearity, respectively, for the average values in regions-of-interest (ROIs). These task-based metrics were used to compare quantitative iodine performance across three CT systems (DSCT, SFCT, and PCCT). LoD, LoQ, and LLLI provide more interpretable metrics for quantitation purposes than metrics based on the pixel standard deviation in ROIs, such as CNR. Notably, it is also considered inappropriate to use CNR as an indicator of image quality for comparisons between systems or reconstructions that cannot be guaranteed to exhibit the same spatial resolution and noise texture [26]. More detailed definitions of LoD, LoQ, and LLLI are provided later in this section.

The analytical pipeline consisted of five steps: (1) repeated scan acquisition across three CT systems using standardized brain and abdominal protocols; (2) ROI-based extraction of iodine signal across slices and repetitions; (3) calibration of measured HU values to iodine concentration using linear regression; (4) derivation of LLLI, LoD, and LoQ using parametric methods inspired by CLSI guidelines [30, 31]; and (5) cross-scanner and cross-protocol comparison, including slice-wise uncertainty estimation and statistical testing.

A noteworthy component of the workflow is a calibration step, in which iodine concentrations (measured in HU values in this study) are mapped to known iodine concentrations. An independent calibration was essential because of variations in the behavior of vendor-provided calibrations across scanners, protocols, and reconstruction algorithms. For our analysis, all systems therefore needed to be evaluated against the same true iodine concentration to enable system- and protocol-agnostic quantification and allow meaningful comparisons.

### Phantom setup

**Fig. 1:**
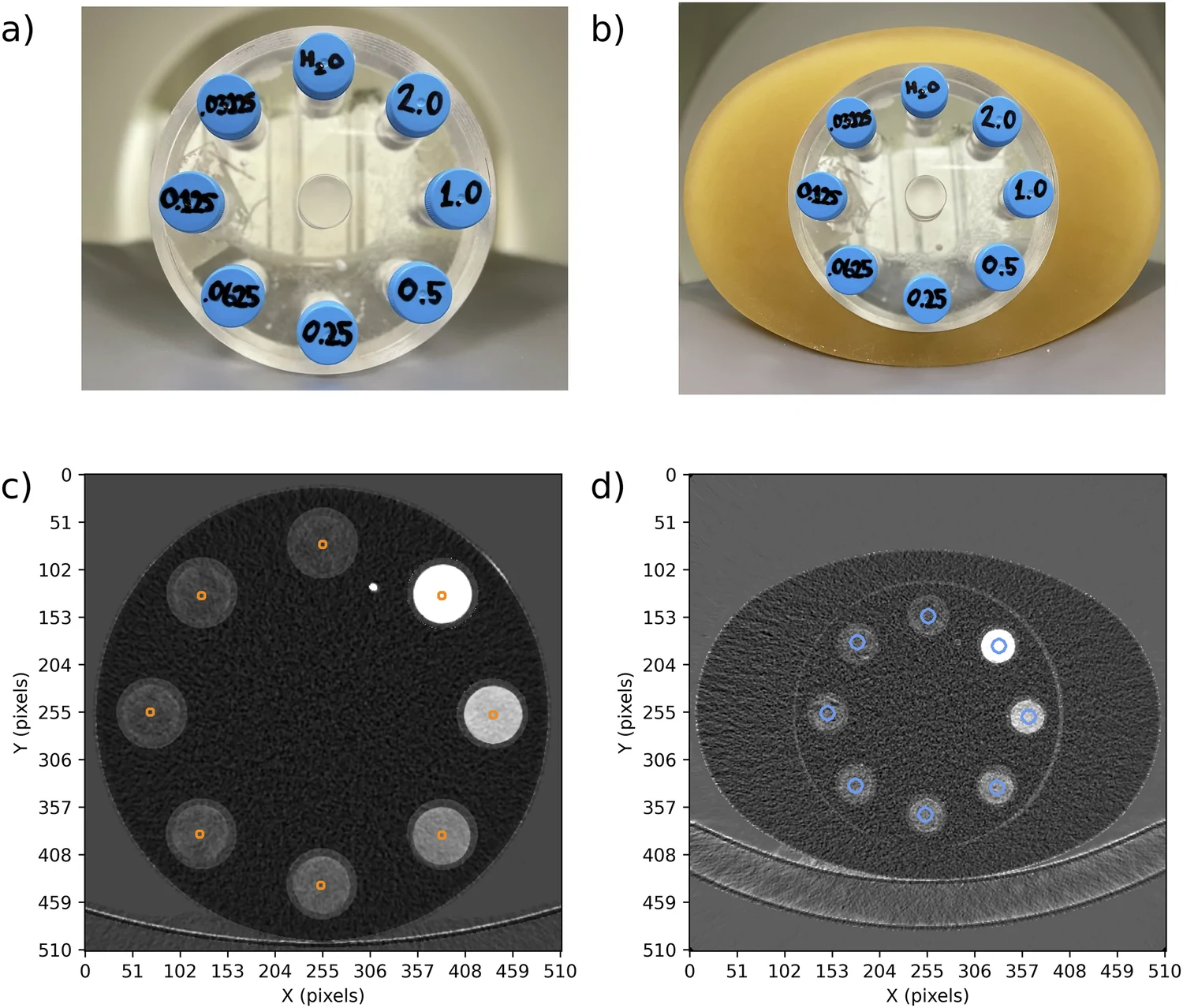
Phantom setup and corresponding iodine images acquired using high-dose protocols. **a** Solid PMMA phantom providing head-equivalent attenuation, with radially positioned tubes containing iodine-water solutions at different concentrations. **b** The same phantom with an additional body annulus to simulate abdomen-equivalent attenuation. **c** Reconstructed iodine image without the body annulus (high-dose brain protocol, FOV 210 mm), showing centrally placed circular ROIs (3 mm in diameter. **d** Reconstructed iodine image of the phantom with the body annulus (high-dose abdominal protocol, FOV 360 mm), showing centrally placed circular ROIs (10 mm in diameter)

To simulate head and abdominal imaging conditions, a custom-built phantom was used: a 15 × 20 cm solid PMMA cylinder with radially drilled holes to accommodate FALCON® conical centrifuge tubes. The tubes were filled with iodine solution prepared by stepwise dilution of Iohexol (Omnipaque™) in distilled water, resulting in concentrations of 2, 1, 0.5, 0.25, 0.125, 0.0625, 0.03125, and 0 mg/mL. This phantom setup approximates the X-ray attenuation characteristics of a human head (Fig. 1a). To simulate an abdominal scenario, it was placed inside an elliptical body annulus (CTP579-15, The Phantom Laboratory, Salem, NY) of dimensions 25 × 35 cm and a thickness of 15 cm (Fig. 1b).

### Scanning protocol and reconstruction

The phantom was scanned using three clinical CT systems from Siemens Healthineers (Forcheim, Germany): a dual-source CT (DSCT; SOMATOM Force, VB20A), a split-filter CT (SFCT; SOMATOM X.ceed, VA40), and a photon-counting CT (PCCT; NAEOTOM Alpha, VB10A). Each system was evaluated under four protocol settings (head and abdominal examination protocol imaged at a low and a high radiation dose). The manufacturers’ recommended protocols (see Table 1) with adjusted CTDI_vol_ values were used. The CTDIvol was set to 3 and 13 mGy for the abdominal protocols and 15 and 50 mGy for the head protocols, to represent typical low-dose and high-dose clinical settings. Each protocol was repeated 20 times per system.

**Table 1:** Protocol and reconstruction parameters

System, protocol	Scan mode	Acquisition (mm)	(kV)	CTDIvol (mGy)	Pitch	Rot. (s)	IR-level
DSCT, abdomen	Spiral dual source	192 × 0.60	100/Sn150	3/13	0.6	0.5	1/2/3/4/5
SFCT, abdomen	Spiral twin beam	64 × 0.60	AuSn 120	3/13	0.45	0.5	1/2/3/4/5
PCCT, abdomen	Spiral quantum plus	144 × 0.40	120	3/13	0.8	0.5	1/2/3/4
DSCT, brain	Spiral dual source	192 × 0.60	80/Sn150	15/50	0.7	1	1/2/3/4/5
SFCT, brain	Spiral twin beam	64 × 0.60	AuSn 120	15/50	0.35	0.5	1/2/3/4/5
PCCT, brain	Spiral quantum plus	144 × 0.40	120	15/50	0.35	0.5	1/2/3/4

*DSCT* Dual-source CT, *IR* Iterative reconstruction, *PCCT* Photon-counting CT, *Rot.* rotation time, *SFCT* Split-filter CT

Iodine maps (in HU), derived from a three-material decomposition model with iodine, fat, and soft tissue as basis materials [34], were reconstructed using a Qr40 kernel, 1.5 slice thickness with 1 mm increment, and a 512 × 512 matrix size. The field of view was set to 210 mm for brain and 360 mm for abdominal protocols. Iterative reconstruction algorithms (QIR for PCCT and ADMIRE for DSCT/SFCT) were applied at varying levels. From each reconstructed volume, 10 slices were selected by extracting every second slice within the central region of the phantom, ensuring non-overlapping slices per repeat. This procedure was applied to all 20 repeats (head/abdomen, low/high radiation dose, IR-levels 1–4 for QIR and 1–5 for ADMIRE) across all three scanners.

### Calibration curve

**Fig. 2:**
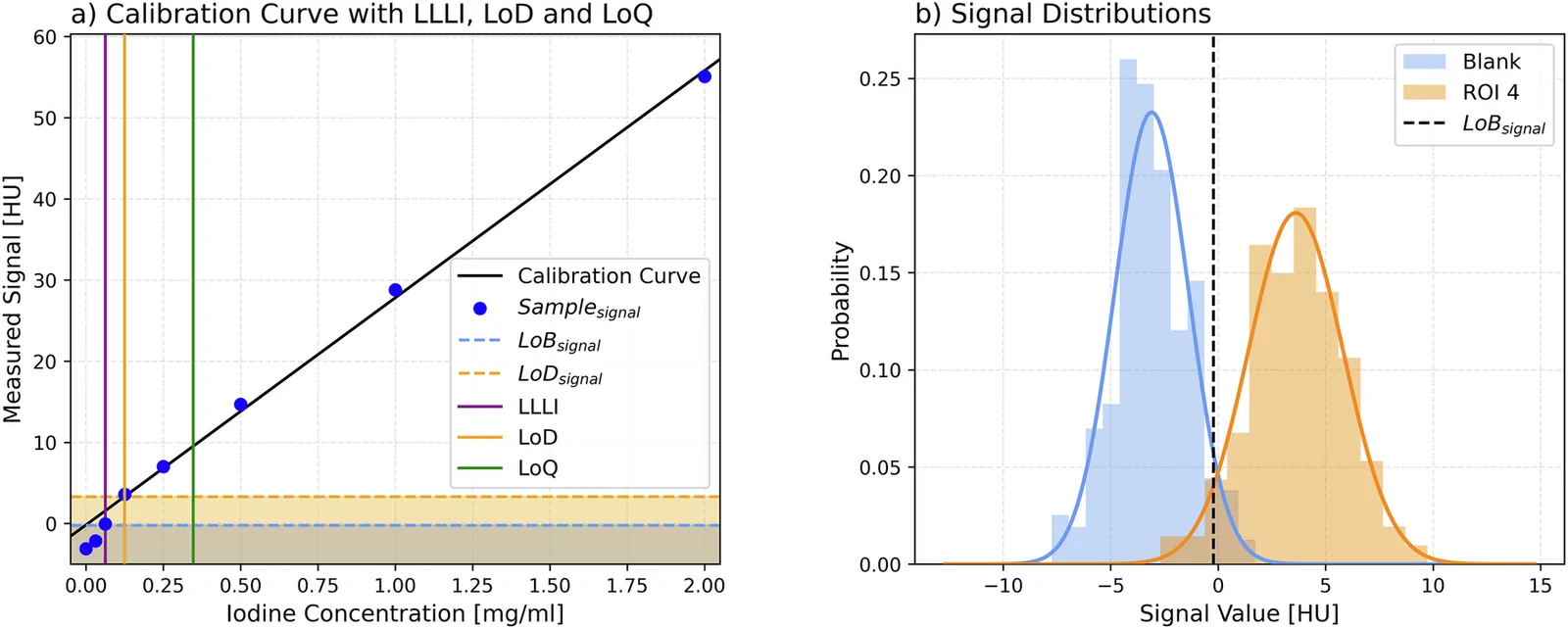
Calibration curve and histogram illustrating LoB and signal overlap. **a** Visualization of calibration curve showing the linear relationship between the measured signal intensity (HU) and the known iodine concentration (mg/mL), with indicated LoB, LoD, LoQ, and LLLI. **b** A representative histogram that illustrates the overlap between the blank and low concentration sample distributions of the measured signal intensity (HU), with the LoB marked as a vertical reference line

For each protocol, calibration curves were established by plotting the measured signal intensities (HU) from ROIs, placed in the center of each test tube (Fig. 1c, d), averaged across all selected slices and 20 repeated acquisitions, against the known iodine concentrations (mg/mL). A linear least squares regression was performed in Python to determine the slope (C_slope_) and intercept (C_int_) of the calibration curve, which were used to convert signal measurements into iodine concentration (see Fig. 2a). The analysis followed the “classical approach” outlined in CLSI guidelines [30]. Prior to applying the parametric methods for calculating the metrics LoD and LoQ, the distribution of ROI measurements from the blank samples was visually inspected. This step was performed to confirm that the assumptions of normality were met, in accordance with CLSI EP17-A2 recommendations.

### Lower limit of the linear interval

This procedure constitutes the formal linearity assessment of the calibration curve and goes beyond visual inspection by applying a predefined statistical acceptance criterion. The lower limit of the interval (LLLI) refers to the lowest iodine concentration at which the system produces values that can be considered directly proportional to the actual concentration [31]. Below this level, the relationship between the measured values and the true values may become inconsistent or unreliable. To determine the LLLI and ensure a statistically robust calibration curve, the following methodology was applied: the lowest concentration data points were progressively excluded and the linear relation refitted until two consecutive points fulfilled a predefined criterion. This criterion required that the 95% confidence interval of a measured concentration, after applying the linear model, fell within a threshold of ± 100% of the nominal concentration. The threshold was investigator-defined and selected empirically after visualization of the data before setting to a fixed value (± 100%). We note that the CLSI EP6 2nd edn guidelines [31] emphasize the use of judgment in the selection of the allowable deviation from linearity and the use of data visualization.

### Limit of blank

In this study, a signal corresponds to the mean of the pixel values in the ROI. The limit of blank (LoB) was calculated independently for each reconstruction setting, protocol, and CT system. LoB is defined as the threshold above which the probability of a false positive signal from a blank sample (0 mg/mL of iodine) within a region of interest (ROI) is below a pre-selected value, accounting for background noise and measurement variability. The LoB is expressed in HU, as the iodine signal was measured in HU from the reconstructed images. In this study, the measurements were obtained by placing an ROI in the center of the respective test tube in each iodine image and taking the mean pixel value. A parametric approach based on the CLSI guidelines [29, 30] was applied to estimate the LoB:

$${LoB}={m}_{0}+{c}_{s}\cdot {s}_{0}$$

where, ${m}_{0}$, is the mean signal from multiple measurements of the blank ROI, ${s}_{0}$ is the corresponding standard deviation, and ${c}_{s}$, is a statistical multiplier that adjusts for both the desired confidence level and the bias in the standard deviation estimation due to small sample sizes. The multiplier ${c}_{s}$ was calculated as:

$${c}_{s}={{z}_{1-\alpha }\left(1-\frac{1}{4(n-1)}\right)}^{-1}$$

Here, ${z}_{1-\alpha },$ is the z-score for a one-sided 95% confidence interval (${z}_{0.95}=\,1.645$), and *n* is the number of repeated measurements per slice. This calculation includes the first two terms of the bias correction factor for small sample sizes, ensuring that the LoB captures the 95th percentile of the blank distribution while accounting for sampling variability and bias [28–30]. Figure 2b illustrates the concept of LoB. The blue histogram represents the distribution from a blank sample (0 mg/mL). The vertical black line marks the LoB, which corresponds to the 95th percentile of the blank distribution. This means that 95% of blank measurements are expected to be below this threshold, while the remaining 5% may exceed it due to random variation.

### Limit of detection

The limit of detection (LoD) is defined as the lowest iodine concentration that could be reliably distinguished from the background noise. It was calculated using repeated measurements across varying iodine concentrations. For each ROI *j*, the signal lower bound, ${P}_{j}$, was computed:

$${P}_{j}={m}_{j}-{c}_{s}\cdot \,{s}_{j}$$

The smallest concentration j* for which the signal ${P}_{j* }\ge {LoB}$ was identified. This corresponds to the lowest concentration where the 5th percentile of the signal exceeds the LoB. Since the standard deviation varies with concentration, the SD at j* was used to ensure an accurate LoD estimate. The corresponding LoD (in mg/mL) was calculated as:

$${LoD}=\frac{{LoB}+{c}_{s}\cdot {s}_{j* }-{C}_{{\mathrm{int}}}}{{C}_{{slope}}}$$

The orange histogram in Fig. 2b represents a low iodine concentration (0.13 mg/mL). The LoD is defined as the lowest concentration at which 95% of the measurements are expected to exceed the LoB. In the figure, this is approximated by the orange distribution, where most values lie to the right of the LoB line. This ensures that the signal from a real (but low) concentration is unlikely to be mistaken for background noise, as illustrated by the overlap with the orange distribution. According to the predefined criteria, LoD values must not fall below the LLLI [30]. In cases where the mean LoD was below this threshold, results were reported as “≤ LLLI”, since the parametric approach is not valid in non-linear regions and such measurements lie outside the validated range.

### Limit of quantification

The limit of quantification (LoQ) is defined as the lowest concentration of iodine at which predefined criteria for accuracy or precision are met. Only data points with nominal concentrations greater than the LLLI were included in the analysis, ensuring that only measurements with a signal reliably above the system’s lower detection threshold are used. The coefficient of variation (CV) was calculated for each concentration, and a precision profile was fitted using a power-law model. The LoQ was interpolated as the concentration corresponding to a target CV of 20%. This approach follows the precision profile method as described in the CLSI EP17-A2 guidelines. By definition, LoQ is equal to or greater than LoD. In cases when LoQ exceeds the maximum calibration concentration (2.0 mg/mL), results are marked with an asterisk to denote extrapolation of the precision profile beyond the characterized range.

### Slice-wise approach to analysis and statistics

The quantitative metrics in this study—LoB, LoD, and LoQ—were calculated slice-wise along the *z*-axis across all repeated images. For each protocol, reconstruction setting, and CT system, measurements were obtained per slice, treating each slice as an independent observation. To mitigate potential inter-slice correlation, every second slice was selected for analysis.

A consistent set of circular ROIs, placed at the center of each vial, was applied across all slices and repeats. The ROIs were predefined with diameters of either 3 mm or 10 mm, depending on the lesion size being simulated. For the purposes of this study, it is assumed that these sizes represent the drawing of an ROI within a small or large lesion, respectively. The final metric values were derived by averaging the slice-specific results to obtain a protocol-level estimate. To quantify the uncertainty in the LoD and LoQ estimates, the standard error of the mean (SE) was calculated across the slice values, and an uncertainty range of ± 2 SE was applied. This coverage factor approximates a 95% confidence interval for the sample sizes (number of slices) in this study.

A one-way Welch ANOVA, followed by a pairwise Games–Howell *post hoc* test, was performed using the Pingouin library in Python, to assess statistically significant differences between the CT systems across protocols. The Games–Howell procedure accounts for unequal variances, sample sizes and provides adjusted *p*-values for multiple pairwise comparisons [35]. No further multiple comparison corrections were applied for other factors (*i.e*., ROI size or protocol).

In addition to lesion-specific measurements, background noise was measured using a 30 mm ROI placed in the center of the phantom; this provides an indication of noise within an image. To compare contrast performance, C_slope_ values were derived from the calibration curve as signal response per mg/mL of iodine.

## Results

Visual inspection of histograms of HU values from ROI measurements in the blank samples revealed no apparent deviations from normality or truncations around zero. This supports the validity of the parametric approach used for calculating LoD and LoQ, in line with CLSI EP17-A2 recommendations.

**Fig. 3:**
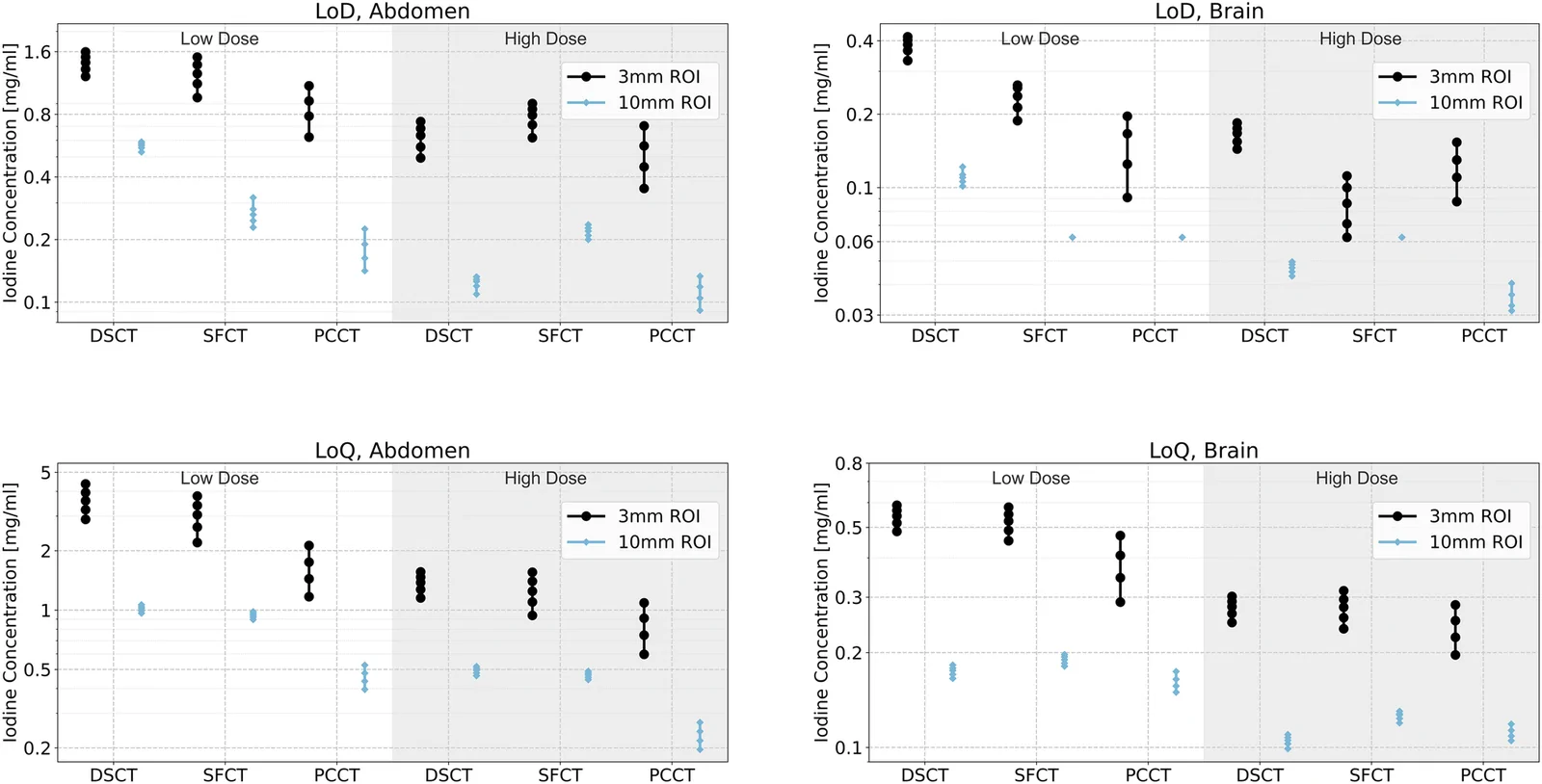
Plot illustrating the LoD and LoQ for the three imaging systems: DSCT, SFCT, and PCCT. Results are shown across IR-levels and all protocol settings, using simulated lesion sizes corresponding to ROIs of 3 mm and 10 mm. The *y*-axis is displayed on a logarithmic scale

The performance of the DSCT, SFCT, and PCCT systems across all protocol settings is illustrated in Fig. 3, showing variations in LoD and LoQ with respect to ROI sizes, radiation doses, protocols, and IR-levels. LoD and LoQ improved with increasing radiation dose for small (3 mm) and large (10 mm) ROIs. The absolute improvements were consistently greater for small ROI sizes across all systems. Relative improvements were generally higher for small ROIs, although not uniformly across all systems and metrics. For large ROI sizes, improvements were still present but comparatively smaller in absolute terms, and system performances converged.

The absolute improvement in both LoD and LoQ with increasing IR-level was greater for small (3 mm) compared to large (10 mm) ROIs. This pattern was observed across all three CT systems (DSCT, SFCT, PCCT), regardless of protocol (high-dose or low-dose). The effect of higher IR-levels generally diminished with increasing radiation dose; this applied to both 3 mm and 10 mm ROIs and was reflected in a reduced absolute improvement in LoD and LoQ. On average, the relative improvement for 3 mm ROIs was also markedly greater than for 10 mm ROIs in terms of both LoD and LoQ, although the differences are not consistent in every individual comparison.

To reflect the typical IR strength used in clinical practice, the results for a medium level of noise suppression (IR-level 3) are presented in Tables 2 and 3. Full results across all IR-levels and protocol combinations are provided in the Supplementary Material (Tables S1 and S2). These tables summarize background noise, C_slope_, and the metrics LoD, LoQ, and LLLI for simulated lesion sizes (3 mm and 10 mm ROIs).

**Table 2:** Quantitative metrics derived from 3 mm ROIs for each system and protocol combination at IR-level 3

3 mm	CTDIvol	SD_BG_	C_slope_	LoD ± 2 SE_LoD_	LoQ ± 2 SE_LoQ_	LLLI
System, IR-level	(mGy)	(HU)	(HU/mg/mL)	(mg I/mL)	(mg I/mL)	(mg I/mL)
DSCT, ADMIRE 3						
Abdominal	3	22	23.2	1.420 ± 0.226	3.589* ± 0.942	0.031
	13	13	22.9	0.634 ± 0.080	1.380 ± 0.122	0.063
Brain	15	8.3	27.4	0.386 ± 0.028	0.544 ± 0.036	0.031
	50	4.9	28.2	0.167 ± 0.015	0.280 ± 0.012	0.031
SFCT, ADMIRE 3						
Abdominal	3	23	21.8	1.255 ± 0.182	3.042* ± 0.515	0.031
	13	14	21.0	0.792 ± 0.116	1.248 ± 0.182	0.031
Brain	15	9.1	24.2	0.237 ± 0.030	0.524 ± 0.031	0.063
	50	5.1	23.9	0.086 ± 0.023	0.279 ± 0.031	0.063
PCCT, QIR 3						
Abdominal	3	14	29.4	0.784 ± 0.052	1.442 ± 0.125	0.031
	13	9.9	28.6	0.447 ± 0.046	0.747 ± 0.040	0.031
Brain	15	5.9	28.0	0.125 ± 0.012	0.346 ± 0.007	0.063
	50	3.5	29.2	0.110 ± 0.016	0.224 ± 0.015	0.031

Values marked with an asterisk (*) exceed the upper limit of iodine concentration (2.0 mg/mL)

*C*_*slope*_ Slope of calibration curve, *DSCT* Dual-source CT, *IR* Iterative reconstruction, *LLLI* Lower limit of the linear interval, *LoD* Limit of detection, *LoQ* Limit of quantification, *PCCT* Photon-counting CT, *ROI* Region of interest, *SD* Standard deviation, *SE* Standard error of the mean, *SFCT* Split-filter CT

**Table 3:** Quantitative metrics derived from 10 mm ROIs for each system and protocol combination at IR-level 3

10 mm	CTDIvol	SD_BG_	C_slope_	LoD ± 2 SE_LoD_	LoQ ± 2 SE_LoQ_	LLLI
System, IR-level	(mGy)	(HU)	(HU/mg/mL)	(mg I/mL)	(mg I/mL)	(mg I/mL)
DSCT, ADMIRE 3						
Abdominal	3	22	23.6	0.566 ± 0.060	1.019 ± 0.040	0.031
	13	13	23.3	0.126 ± 0.030	0.496 ± 0.024	0.063
Brain	15	8.3	27.8	0.110 ± 0.011	0.176 ± 0.011	0.031
	50	4.9	28.1	0.047 ± 0.004	0.105 ± 0.006	0.031
SFCT, ADMIRE 3						
Abdominal	3	23	20.6	0.263 ± 0.033	0.941 ± 0.088	0.063
	13	14	20.4	0.220 ± 0.041	0.469 ± 0.046	0.063
Brain	15	9.1	23.9	≤ LLLI	0.190 ± 0.016	0.063
	50	5.1	23.8	≤ LLLI	0.127 ± 0.019	0.063
PCCT, QIR 3						
Abdominal	3	14	29.4	0.163 ± 0.025	0.435 ± 0.025	0.031
	13	9.9	29.0	0.105 ± 0.023	0.218 ± 0.016	0.031
Brain	15	5.9	28.6	≤ LLLI	0.157 ± 0.008	0.063
	50	3.5	29.6	0.033 ± 0.003	0.109 ± 0.004	0.031

LoD values below the LLLI are presented as ≤ LLLI

*C*_*slope*_ Slope of calibration curve, *DSCT* Dual-source CT, *IR* Iterative reconstruction, *LLLI* Lower limit of the linear interval, *LoD* Limit of detection, *LoQ* Limit of quantification, *PCCT* Photon-counting CT, *ROI* Region of interest, *SDBG* Standard deviation of the background, *SE* Standard error of the mean, *SFCT* Split-filter CT

PCCT consistently demonstrated the highest C_slope_ and lowest noise. Both DSCT and SFCT displayed comparable noise levels, but DSCT exhibited a higher signal response.

**Fig. 4:**
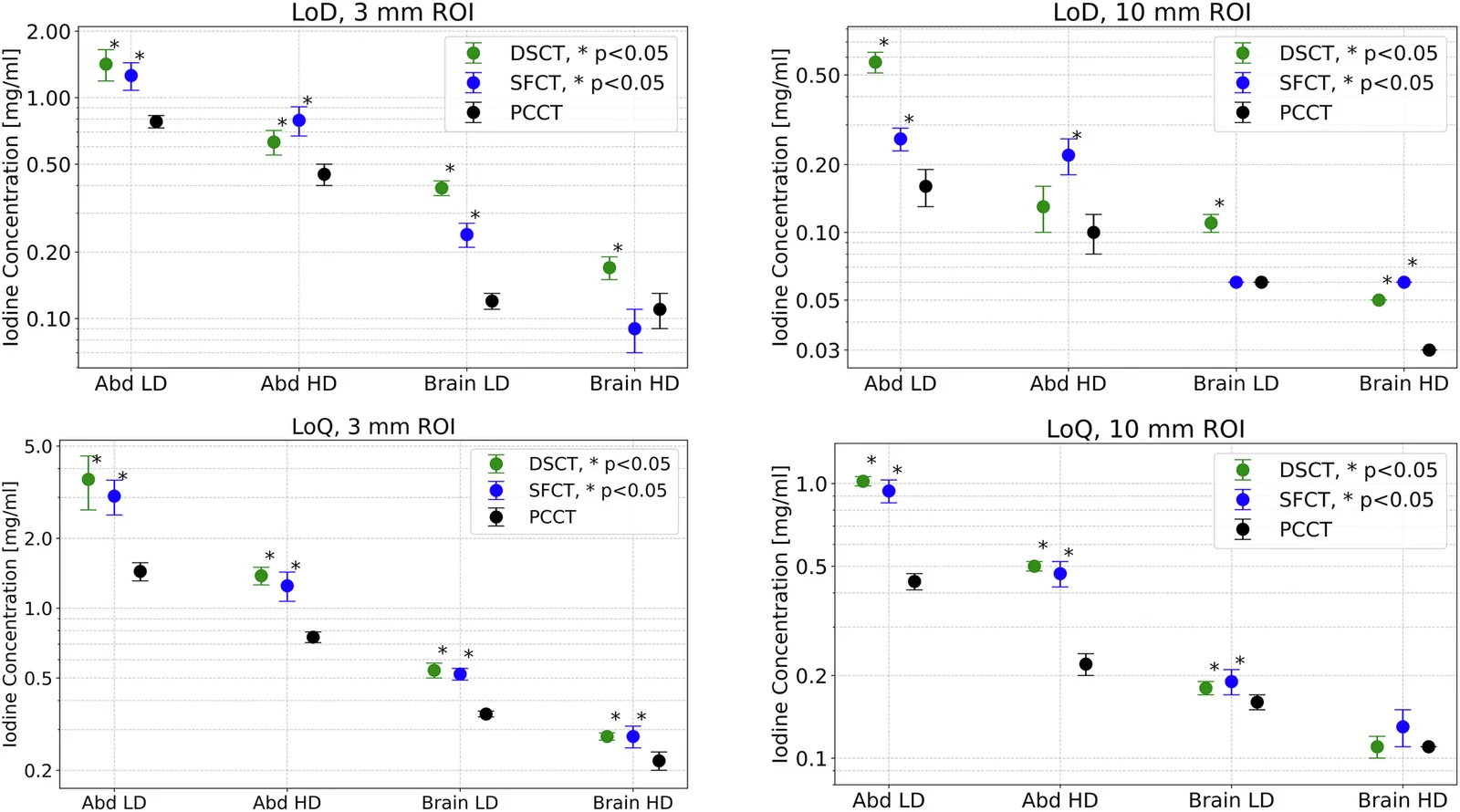
Mean LoD and LoQ across system, protocols, and ROI sizes at IR-level 3. Mean LoD and LoQ values for each system across imaging protocols and simulated lesion sizes (3 mm and 10 mm ROIs) at IR-level 3. Error bars represent ± 2 SE. Statistically significant differences from the PCCT system are marked with asterisks (*), based on Welch’s ANOVA and Games–Howell *post hoc* test. LoD values below the LLLI are replaced by the respective LLLI value for visualization. The *y*-axis is displayed on a logarithmic scale

A visual summary of the LoD and LoQ results for the three scanners, two dose levels, two ROI sizes, and medium IR-level is shown in Fig. 4. PCCT showed significantly lower LoD and LoQ than both DECT systems across most protocols and ROI sizes.

At IR-level 3, for 3 mm ROIs, PCCT demonstrated significantly lower LoD across all protocols (all *p* ≤ 0.003), except in the high-dose brain protocol, where LoD showed comparable results with SFCT. LoQ values were also significantly lower across all four protocols (all *p* ≤ 0.003), including SFCT in the high-dose brain protocol (*p* = 0.019), although with less pronounced significance.

For 10 mm ROIs, PCCT showed significantly lower LoD values in the low-dose abdominal and high-dose brain protocols (*p* ≤ 0.001), while performance was comparable to SFCT in the high-dose abdominal and to DSCT in the low-dose brain protocol. Regarding LoQ, PCCT outperformed both DECT systems in the abdominal and low-dose brain protocols (all *p* < 0.001, except for DSCT low-dose brain with *p* = 0.034 and SFCT with low-dose Brain *p* = 0.006). No significant difference in LoQ was observed between the PCCT and DECT systems in the high-dose brain protocol. The corresponding numerical values and uncertainty estimates are also reported in Tables 2 and 3, while full lists of *p*-values are provided in the Supplementary Material (Tables S3 and S4).

## Discussion

Contrast-to-noise ratio remains a useful and widely applied metric in CT, especially when comparisons are made under similar imaging and reconstruction conditions. However, for cross-system or cross-reconstruction comparisons where spatial resolution and noise texture may differ, task-specific metrics such as LoD and LoQ may provide advantages. Rather than replacing conventional image quality measures, LoD and LoQ are intended to supplement them by quantifying these task-specific thresholds and providing a clinically interpretable link between detectable iodine concentrations and CT system performance.

Quantitative iodine imaging is clinically relevant in multiple scenarios. In abdominal imaging, DECT-based iodine quantification has been shown to differentiate benign from malignant renal lesions using 0.5 mg/mL thresholds [36]. Measurements of iodine density values in stroke patients reported a difference between ischemic and non-ischemic tissue of approximately 0.3 mg/mL [37], and traumatic contusions show leakage ranging from 0.1 to 1.0 mg/mL [38]. For such tasks, the key question is not simply how images appear, but at what iodine concentration a lesion can first be detected and reliably quantified. Linking quantification of a contrast-enhanced lesion (the task) to the level at which it can be reliably detected and quantified is essential.

PCCT demonstrated substantially lower detection and quantification limits than DSCT and SFCT, primarily under low-dose protocols and small ROI sizes. The mechanisms behind these improvements are likely multifactorial and related to enhanced signal response, reduced image noise, and improved scan-to-scan measurement consistency. At higher dose levels or for larger ROIs, performance converged between scanners. This suggests a plateau effect, where benefits from PCCT diminish. LoD and LoQ determinations are influenced not only by quantum noise but also by factors such as fluctuations of the system that may depend on calibration stability and the encroachment of the relevant concentration levels on the lower limit of linear interval (LLLI). These become increasingly important as the quantum noise is suppressed by a higher dose or a larger ROI size.

Under low-dose abdominal protocols, both DSCT and SFCT showed LoQ values that exceeded the upper calibration range (2 mg/mL) for 3 mm ROIs, indicating high variability and reduced repeatability. This suggests that quantitative iodine measurements in small lesions or ROIs and low-dose conditions should be interpreted with caution. In contrast, PCCT maintained relatively lower LoQ values, suggesting better quantifiability even under challenging low-dose conditions. This highlights practical performance differences between detector technologies.

Iterative reconstruction is expected to affect LoD and LoQ primarily through the suppression of image noise. Accordingly, increasing the IR-level substantially improved LoD and LoQ for small ROIs and low-dose acquisitions, where pixel-to-pixel noise still contributes significantly to measurement variability. For larger ROIs at higher radiation doses, where such noise-related variability is already reduced, LoD and LoQ become increasingly governed by system repeatability and stability, resulting in a more limited impact of higher IR-levels.

In line with the observed improvements in LoD and LoQ, PCCT also consistently exhibited the highest contrast response and lowest image noise. Interestingly, despite DSCT showing higher signal and slightly lower noise than SFCT in individual slice measurements, this did not always translate into improved LoD or LoQ. SFCT could even in some cases offer a more stable signal between scans, although the per-slice noise was higher.

While previous PCCT studies have primarily focused on measures such as in-slice image noise, CNR, or qualitative assessments, the present work explicitly accounts for all sources of scan-to-scan variation in mean signal in an ROI—an important factor for quantitative imaging—as well as controlling for differences in noise texture between systems.

Improvements in LoD and LoQ with PCCT compared to DECT systems may have important clinical implications. Under conditions resembling the phantom experiments, they suggest the potential for detecting and quantifying similar lesions with less contrast media, or smaller ROIs with the same radiation dose. This may support safer imaging, particularly for patients with renal impairment. In addition, improved LoD and LoQ at lower dose levels indicate the potential to reduce radiation dose while preserving performance in the detection and quantitation of iodine for a given diagnostic task. Previous clinical studies have demonstrated that contrast volume can be reduced by approximately 17–30% in thoraco-abdominal and abdominal examinations using the first generation of PCCT, without negatively affecting image quality [21, 39]. Although our study did not directly assess contrast dose reduction, the observed improvements in LoD and LoQ are in line with previous findings. While previous studies demonstrated the feasibility of iodine contrast reduction with PCCT in clinical settings, the present work provides a quantitative framework that helps explain these observations by linking detectability and quantifiability to task-specific imaging conditions.

Few previous studies have applied the concepts of LoD and LoQ to quantitation in CT [33, 40–43]. Jacobsen et al [33, 43] did apply LoD and LoQ to DECT and SFCT systems. While their LoQ was based on a coefficient of variation of 20%, this study uses the precision profile approach to interpolate between measured iodine concentrations [30]. In addition, differences in acquisition and reconstruction parameters, as well as the use of a different SFCT system, complicate direct comparisons. Nevertheless, the LoD and LoQ values observed in the present study fall within the same order of magnitude, and the systems’ contrast responses (C_slope_) were comparable.

This study has several limitations. The phantom-based design represents a simplified imaging environment and does not account for tissue heterogeneity and anatomical complexity encountered *in vivo*. A limitation of the slice-wise analysis is that the included slices cannot be assumed to be fully independent, since they originate from overlapping reconstructions within the same helical acquisition. Although every second slice was selected to reduce inter-slice dependence, some residual correlation may remain. The reported standard errors and confidence intervals may therefore be underestimated. In addition, the evaluation was limited to CT systems from a single vendor, which may limit generalizability but allowed a controlled comparison across different detector technologies. The study further relied on vendor-recommended protocols and evaluated only two ROI sizes across a limited range of dose levels. Nevertheless, the use of water-based iodine solutions provides a controlled and reproducible approximation of clinical iodine imaging. The presented results should be interpreted as baseline estimates of detection and quantification performance. The relevance of these metrics may be further explored in more complex phantoms and *in vivo* settings, while acknowledging that increased anatomical and physiological complexity may introduce additional sources of variability beyond system performance.

In summary, we have made the case for the utility of the LoD and LoQ concepts in quantitative imaging using iodine maps and applied them to the case of intercomparisons between two DECT and a PCCT system. The PCCT scanner demonstrated superior performance in the detection and quantification of low iodine concentrations, particularly for small ROIs and low-dose imaging tasks. From a clinical perspective, these findings suggest an improved detection and quantification performance for subtle iodine enhancement on iodine maps under these controlled conditions and may support future protocol optimization strategies, such as iodine contrast dose reduction or radiation dose reduction, pending clinical validation.

## Supplementary information

**Additional file: Table S1.** Quantitative metrics derived from 3 mm ROIs for each system and protocol combination across all IR-levels. **Table S2.** Quantitative metrics derived from 10 mm ROIs for each system and protocol combination across all IR-levels. **Table S3.** *P*-values for 3 mm ROIs comparing PCCT with DECT and SFCT. **Table S4.** *P*-values for 10 mm ROIs comparing PCCT with DECT and SFCT.

## Data Availability

The datasets analyzed during the current study are available from the corresponding author upon reasonable request.

## Abbreviations

CNR: Contrast-to-noise ratio
C_slope_: Slope of calibration curve
CLSI: Clinical and Laboratory Standards Institute
DECT: Dual-energy CT
DSCT: Dual-source CT
HU: Hounsfield unit
IR: Iterative reconstruction
LLLI: Lower limit of the linear interval
LoB: Limit of blank
LoD: Limit of detection
LoQ: Limit of quantification
PCCT: Photon-counting CT
ROI: Region of interest
SD: Standard deviation
SD_BG_: Standard deviation of the background
SE: Standard error of the mean
SFCT: Split-filter CT
