# Supplementary material for: Evaluation of iodine detection and quantification limits in photon-counting CT—a phantom study
Source: Eur Radiol Exp. 2026 Oct 12;10:154. doi: 10.1186/s41747-026-00824-w (PMC13658709; doi:10.1186/s41747-026-00824-w)
Supplement: Supplementary file 1 [file 41747_2026_824_MOESM1_ESM.pdf]

Evaluation of iodine detection and quantification limits in photon counting CT – A phantom study

Electronic supplementary material

Table S1. Quantitative metrics derived from 3 mm ROIs for each system and protocol combination across all IR-levels.

System	IR-Level	CTDI [mGy]	Cslope [HU/mg/ml]	Cint [HU]	LLLI [mg I/mL]	LoD ± 2 x SE [mg I/mL]	LoQ ± 2 x SE [mg I/mL]
DSCT	1	3	23.3	-2.3	0.031	1.596 ± 0.245	4.363* ± 1.305
DSCT	2	3	23.2	-2.3	0.031	1.506 ± 0.237	3.945* ±1.106
DSCT	3	3	23.2	-2.3	0.031	1.420 ± 0.226	3.589* ± 0.942
DSCT	4	3	23.2	-2.3	0.031	1.318 ± 0.209	3.229* ± 0.796
DSCT	5	3	23.2	-2.3	0.031	1.217 ± 0.196	2.885* ±0.651
DSCT	1	13	22.9	-2.3	0.063	0.738 ± 0.095	1.562 ± 0.190
DSCT	2	13	22.9	-2.3	0.063	0.684 ±0.086	1.469 ± 0.153
DSCT	3	13	22.9	-2.3	0.063	0.634 ± 0.080	1.380 ± 0.122
DSCT	4	13	23.0	-2.3	0.063	0.557 ± 0.069	1.272 ± 0.091
DSCT	5	13	23.0	-2.4	0.063	0.493 ± 0.057	1.153 ± 0.070
DSCT	1	15	27.4	0.8	0.031	0.416 ± 0.032	0.588 ± 0.040
DSCT	2	15	27.4	0.8	0.031	0.403 ± 0.029	0.566 ± 0.037
DSCT	3	15	27.4	0.8	0.031	0.386 ± 0.028	0.544 ± 0.036
DSCT	4	15	27.4	0.8	0.031	0.365 ± 0.024	0.517 ± 0.035
DSCT	5	15	27.4	0.8	0.031	0.332 ± 0.025	0.486 ± 0.032
DSCT	1	50	28.2	-0.6	0.031	0.184 ± 0.016	0.302 ± 0.014
DSCT	2	50	28.2	-0.6	0.031	0.175 ± 0.016	0.291 ± 0.013
DSCT	3	50	28.2	-0.6	0.031	0.167 ± 0.015	0.280 ± 0.012
DSCT	4	50	28.2	-0.6	0.031	0.154 ± 0.016	0.266 ± 0.011
DSCT	5	50	28.2	-0.7	0.031	0.144 ± 0.015	0.249 ± 0.012
PCCT	1	3	29.3	1.4	0.031	1.096 ± 0.095	2.126* ± 0.217
PCCT	2	3	29.3	1.3	0.031	0.927 ± 0.047	1.750 ± 0.173
PCCT	3	3	29.4	1.3	0.031	0.784 ± 0.052	1.442 ± 0.125
PCCT	4	3	29.4	1.3	0.031	0.621 ± 0.060	1.169 ± 0.085
PCCT	1	13	28.5	0.6	0.031	0.704 ± 0.041	1.089 ± 0.075
PCCT	2	13	28.5	0.5	0.031	0.564 ± 0.059	0.910 ± 0.053
PCCT	3	13	28.6	0.4	0.031	0.447 ± 0.046	0.747 ± 0.040
PCCT	4	13	28.6	0.3	0.031	0.352 ± 0.041	0.596 ± 0.034
PCCT	1	15	27.9	-0.1	0.063	0.196 ± 0.021	0.471 ± 0.013
PCCT	2	15	27.9	-0.1	0.063	0.166 ± 0.021	0.408 ± 0.009
PCCT	3	15	28.0	-0.2	0.063	0.125 ± 0.012	0.346 ± 0.007
PCCT	4	15	28.0	-0.2	0.063	0.091 ± 0.012	0.289 ± 0.007
PCCT	1	50	29.3	-2.5	0.031	0.153 ± 0.024	0.284 ± 0.021
PCCT	2	50	29.3	-2.5	0.031	0.130 ± 0.018	0.253 ± 0.018
PCCT	3	50	29.3	-2.5	0.031	0.110 ± 0.016	0.224 ± 0.015

PCCT	4	50	29.2	-2.5	0.031	0.088 ± 0.013	0.197 ± 0.012
SFCT	1	3	22.0	-10.8	0.031	1.506 ± 0.205	3.788* ± 0.762
SFCT	2	3	21.9	-10.6	0.031	1.385 ± 0.187	3.397* ± 0.639
SFCT	3	3	21.8	-10.4	0.031	1.255 ± 0.182	3.042* ± 0.515
SFCT	4	3	21.7	-10.2	0.031	1.123 ± 0.155	2.638* ± 0.384
SFCT	5	3	21.6	-10.0	0.031	0.962 ± 0.134	2.201* ± 0.253
SFCT	1	13	21.1	-7.3	0.031	0.902 ± 0.128	1.556 ± 0.262
SFCT	2	13	21.0	-7.3	0.031	0.848 ± 0.123	1.399 ± 0.221
SFCT	3	13	21.0	-7.2	0.031	0.792 ±0.116	1.248 ± 0.182
SFCT	4	13	20.9	-7.2	0.031	0.712 ± 0.102	1.100 ± 0.152
SFCT	5	13	20.8	-7.2	0.031	0.618 ± 0.105	0.939 ± 0.124
SFCT	1	15	24.2	1.9	0.063	0.263 ± 0.026	0.580 ±0.034
SFCT	2	15	24.2	1.9	0.063	0.257 ± 0.031	0.551 ± 0.033
SFCT	3	15	24.2	1.9	0.063	0.237 ± 0.030	0.524 ± 0.031
SFCT	4	15	24.2	1.9	0.063	0.213 ± 0.032	0.490 ± 0.030
SFCT	5	15	24.2	1.8	0.063	0.188 ± 0.030	0.453 ± 0.029
SFCT	1	50	24.0	2.6	0.063	0.112 ± 0.028	0.315 ± 0.031
SFCT	2	50	24.0	2.6	0.063	0.100 ± 0.027	0.296 ± 0.032
SFCT	3	50	24.0	2.6	0.063	0.086 ± 0.023	0.279 ± 0.031
SFCT	4	50	23.9	2.6	0.063	0.071 ± 0.023	0.258 ± 0.030
SFCT	5	50	23.9	2.6	0.063	≤ LLLI	0.238 ± 0.029

Values marked with asterisk (*) exceed the upper limit of iodine concentration (2.0 mg/ml). LoD values below the LLLI are presented as ≤ LLLI. Abbreviations: C_{int} = Intercept of calibration curve; C_{slope} = Slope of calibration curve; DSCT = Dual-source CT; IR = Iterative reconstruction; LLLI = Lower limit of the linear interval; LoD = Limit of detection; LoQ = Limit of quantification; PCCT = Photon-counting CT; ROI = Region of interest; SD_{BG} = Standard deviation of the background; SE = Standard error of the mean; SFCT = Split-filter CT.

Legend: Cslope, Cint, LoD, LoQ and LLLI were calculated using 3 mm ROIs. Background noise was measured using 30 mm ROIs. Values are presented with expanded uncertainty (±2 SE).

Table S2. Quantitative metrics derived from 10 mm ROIs for each system and protocol combination across all IR-levels.

System	IR-Level	CTDI [mGy]	Cslope [HU/mg/ml]	Cint [HU]	LLLI [mg I/mL]	LoD ± 2 x SE [mg I/mL]	LoQ ± 2 x SE [mg I/mL]
DSCT	1	3	23.6	-3.3	0.031	0.590 ± 0.060	1.064 ± 0.022
DSCT	2	3	23.6	-3.3	0.031	0.578 ± 0.061	1.040 ± 0.021
DSCT	3	3	23.6	-3.3	0.031	0.566 ± 0.060	1.019 ± 0.020
DSCT	4	3	23.6	-3.3	0.031	0.552 ± 0.058	0.995 ± 0.019
DSCT	5	3	23.6	-3.3	0.031	0.527 ± 0.055	0.965 ± 0.018
DSCT	1	13	23.2	-2.8	0.063	0.133 ± 0.032	0.518 ± 0.013
DSCT	2	13	23.2	-2.8	0.063	0.129 ± 0.031	0.507 ± 0.012
DSCT	3	13	23.3	-2.8	0.063	0.126 ± 0.030	0.496 ± 0.012
DSCT	4	13	23.3	-2.8	0.063	0.120 ± 0.029	0.480 ± 0.011
DSCT	5	13	23.3	-2.8	0.063	0.109 ± 0.022	0.465 ± 0.011
DSCT	1	15	27.8	0.7	0.031	0.122 ± 0.008	0.183 ± 0.006
DSCT	2	15	27.8	0.7	0.031	0.113 ± 0.011	0.179 ± 0.006
DSCT	3	15	27.8	0.7	0.031	0.110 ± 0.011	0.176 ± 0.005
DSCT	4	15	27.8	0.7	0.031	0.106 ± 0.010	0.171 ± 0.005
DSCT	5	15	27.8	0.7	0.031	0.101 ± 0.010	0.166 ± 0.005
DSCT	1	50	28.1	-0.7	0.031	0.050 ± 0.005	0.110 ± 0.003
DSCT	2	50	28.1	-0.7	0.031	0.048 ± 0.005	0.107 ± 0.003
DSCT	3	50	28.1	-0.7	0.031	0.047 ± 0.004	0.105 ± 0.003
DSCT	4	50	28.1	-0.7	0.031	0.045 ± 0.002	0.103 ± 0.003
DSCT	5	50	28.1	-0.7	0.031	0.043 ± 0.004	0.099 ± 0.002
PCCT	1	3	29.4	1.3	0.031	0.225 ± 0.024	0.526 ± 0.016
PCCT	2	3	29.4	1.3	0.031	0.190 ± 0.027	0.479 ± 0.014
PCCT	3	3	29.4	1.3	0.031	0.163 ± 0.025	0.435 ± 0.013
PCCT	4	3	29.4	1.2	0.031	0.141 ± 0.032	0.396 ± 0.012
PCCT	1	13	29.0	0.3	0.031	0.133 ± 0.021	0.269 ± 0.010
PCCT	2	13	29.0	0.2	0.031	0.119 ± 0.021	0.242 ± 0.009

PCCT	3	13	29.0	0.2	0.031	0.105 ± 0.023	0.218 ± 0.008
PCCT	4	13	29.0	0.2	0.031	0.091 ± 0.021	0.196 ± 0.008
PCCT	1	15	28.6	-0.3	0.063	≤ LLLI	0.174 ± 0.005
PCCT	2	15	28.6	-0.3	0.063	≤ LLLI	0.165 ± 0.005
PCCT	3	15	28.6	-0.3	0.063	≤ LLLI	0.157 ± 0.004
PCCT	4	15	28.6	-0.3	0.063	≤ LLLI	0.150 ± 0.003
PCCT	1	50	29.6	-2.3	0.031	0.041 ± 0.004	0.119 ± 0.003
PCCT	2	50	29.6	-2.3	0.031	0.036 ± 0.003	0.113 ± 0.002
PCCT	3	50	29.6	-2.3	0.031	0.033 ± 0.003	0.109 ± 0.002
PCCT	4	50	29.6	-2.3	0.031	≤ LLLI	0.105 ± 0.002
SFCT	1	3	20.6	-8.8	0.063	0.318 ± 0.043	0.981 ± 0.045
SFCT	2	3	20.6	-8.6	0.063	0.280 ± 0.035	0.960 ± 0.044
SFCT	3	3	20.6	-8.5	0.063	0.263 ± 0.033	0.941 ± 0.044
SFCT	4	3	20.5	-8.3	0.063	0.246 ± 0.031	0.919 ± 0.042
SFCT	5	3	20.5	-8.1	0.063	0.229 ±0.030	0.896 ± 0.039
SFCT	1	13	20.4	-6.5	0.063	0.236 ± 0.043	0.490 ± 0.025
SFCT	2	13	20.4	-6.5	0.063	0.227 ± 0.042	0.478 ± 0.023
SFCT	3	13	20.4	-6.4	0.063	0.220 ± 0.041	0.469 ± 0.023
SFCT	4	13	20.3	-6.4	0.063	0.209 ± 0.040	0.456 ± 0.022
SFCT	5	13	20.3	-6.3	0.063	0.200 ± 0.040	0.444 ± 0.021
SFCT	1	15	23.9	2.2	0.063	≤ LLLI	0.197 ± 0.008
SFCT	2	15	23.9	2.1	0.063	≤ LLLI	0.194 ± 0.008
SFCT	3	15	23.9	2.1	0.063	≤ LLLI	0.190 ± 0.008
SFCT	4	15	23.9	2.1	0.063	≤ LLLI	0.185 ± 0.008
SFCT	5	15	23.9	2.0	0.063	≤ LLLI	0.181 ± 0.008
SFCT	1	50	23.8	2.9	0.063	≤ LLLI	0.130 ± 0.010
SFCT	2	50	23.8	2.9	0.063	≤ LLLI	0.128 ± 0.010
SFCT	3	50	23.8	2.9	0.063	≤ LLLI	0.127 ± 0.010
SFCT	4	50	23.8	2.9	0.063	≤ LLLI	0.124 ± 0.009
SFCT	5	50	23.8	2.8	0.063	≤ LLLI	0.120 ± 0.010

LoD values below the LLLI are presented as ≤ LLLI. Abbreviations: C_{int} = Intercept of calibration curve; C_{slope} = Slope of calibration curve; DSCT = Dual-source CT; IR = Iterative reconstruction; LLLI = Lower limit of the linear interval; LoD = Limit of detection; LoQ = Limit of quantification; PCCT = Photon-counting CT; ROI = Region of interest; SD_{BG} = Standard deviation of the background; SE = Standard error of the mean; SFCT = Split-filter CT.

Legend: Cslope, Cint, LoD, LoQ and LLLI were calculated using 10 mm ROIs. Background noise was measured using 30 mm ROIs. Values are presented with expanded uncertainty (±2 SE).

Table S3. P-values for 3 mm ROIs comparing PCCT with DECT and SFCT.

Protocol	CTDI [mGy]	LoD, DECT	LoD, SFCT	LoQ, DECT	LoQ, SFCT
LD Abdomen	3	0.001	0.001	0.003	0.000
HD Abdomen	13	0.003	0.000	0.000	0.001
LD Brain	15	0.000	0.000	0.000	0.000
HD Brain	50	0.000	0.221	0.000	0.019

Abbreviations: DSCT = Dual-source CT; HD = High-dose; LD = Low-dose; PCCT = Photon-counting CT; ROI = Region of interest; SFCT = Split-filter CT.

Legend: All reconstructions were performed using IR-level 3. P-values were obtained from pairwise Games-Howell tests and are reported with three decimals.

Table S4. P-values for 10 mm ROIs comparing PCCT with DECT and SFCT.

Protocol	CTDI [mGy]	LoD, DECT	LoD, SFCT	LoQ, DECT	LoQ, SFCT
LD Abdomen	3	0.000	0.000	0.000	0.000
HD Abdomen	13	0.516	0.001	0.000	0.000
LD Brain	15	0.000	–	0.034	0.006
HD Brain	50	0.000	0.000	0.598	0.211

(–) indicates not applicable due to identical LoD values (LoD = LLLI). Abbreviations: DSCT = Dual-source CT; HD = High-dose; LD = Low-dose; PCCT = Photon-counting CT; ROI = Region of interest; SFCT = Split-filter CT.

Legend: All reconstructions were performed using IR-level 3. P-values were obtained from pairwise Games-Howell tests and are reported with three decimals.
